# The longitudinal impact of health behaviours on mental health, diabetes distress, and quality of life in people with type 1, type 2, and gestational diabetes: A scoping review

**DOI:** 10.1192/j.eurpsy.2025.1208

**Published:** 2025-08-26

**Authors:** A. M. McInerney, C. Flynn, K. Fardfini-Ruginets, S. Liu, C. Koretsidou, R. J. Burns, S. S. Deschênes

**Affiliations:** 1 Psychology, University College Dublin, Dublin, Ireland; 2 Pobulation-Based Medicine, Eberhard Karls Universität Tübingen, Tübingen, Germany; 3 Psychology, Carleton University, Ottawa, Canada; 4 Department of Medical Epidemiology and Biostatistics, Karolinska Institute, Solna, Sweden

## Abstract

**Introduction:**

There can be a considerable mental health burden to living with diabetes. Health behaviours are modifiable factors that influence mental health in the general population. However, despite the centrality of health behaviours to diabetes management, there are significant gaps in our understanding of their longitudinal impact on mental health in people with diabetes.

**Objectives:**

This scoping review aimed to synthesise the longitudinal evidence from observational and intervention research on the impact of health behaviours on mental health and related psychological factors in people with type 1, type 2, and gestational diabetes.

**Methods:**

PubMed, PsychINFO, Embase, CINAHL, and PsycArticles were searched for intervention and observational studies examining the effect of health-promoting, health-risk, or diabetes-specific health behaviours on aspects of mental health, diabetes distress, and quality of life, in people with all types of diabetes. Abstracts, titles, and full texts were screened by two independent reviewers.

**Results:**

In total, 100 relevant studies were identified, including 29 observational studies and 71 intervention studies. Studies had a mean follow-up time of 12.9 ± 17.8 months. The health behaviours investigated in the included studies were adherence, alcohol, carbohydrate-counting, diet, diet and exercise combined, exercise, fasting, medical visits, sleep, self-monitoring blood glucose, smoking, and weight. The review identified knowledge gaps surrounding diabetes-specific behaviours, behaviour interactions/clusters, sleep, sedentary behaviour, screen-time, gestational diabetes, mood and ecologically valid and objective measurements. Exercise was the most freqently investigated health behaviour and also the most likely to be found to have a mental health promoting impact.

**Conclusions:**

Findings suggest that health-promoting behaviours influence mental health in people with diabetes. There is less conclusive evidence regarding the impact of health-risk or diabetes-specific health behaviours. In particular, a broad range of physical activities may improve mental health and wellbeing in people with diabetes.

**Disclosure of Interest:**

None Declared

